# Will Global Climate Change Alter Fundamental Human Immune Reactivity: Implications for Child Health?

**DOI:** 10.3390/children1030403

**Published:** 2014-11-11

**Authors:** Ashwin Swaminathan, Robyn M. Lucas, David Harley, Anthony J. McMichael

**Affiliations:** 1 National Centre for Epidemiology and Population Health, Australian National University, Corner of Mills and Eggleston Roads, Canberra, Australian Capital Territory 2601, Australia; E-Mails: robyn.lucas@anu.edu.au; david.harley@anu.edu.au; 2 Departments of General Medicine and Infectious Diseases, Canberra Hospital, Yamba Drive, Garran, Canberra, Australian Capital Territory 2605, Australia; 3 Australian National University Medical School, Australian National University, Canberra, Australian Capital Territory 0200, Australia; 4 Telethon Kids Institute, University of Western Australia, 100 Roberts Road, Subiaco, Perth, Western Australia 6008, Australia

**Keywords:** children, paediatrics, climate change, immunity, ultraviolet radiation, heat stress, psychological stress, malnutrition

## Abstract

The human immune system is an interface across which many climate change sensitive exposures can affect health outcomes. Gaining an understanding of the range of potential effects that climate change could have on immune function will be of considerable importance, particularly for child health, but has, as yet, received minimal research attention. We postulate several mechanisms whereby climate change sensitive exposures and conditions will subtly impair aspects of the human immune response, thereby altering the distribution of vulnerability within populations—particularly for children—to infection and disease. Key climate change-sensitive pathways include under-nutrition, psychological stress and exposure to ambient ultraviolet radiation, with effects on susceptibility to infection, allergy and autoimmune diseases. Other climate change sensitive exposures may also be important and interact, either additively or synergistically, to alter health risks. Conducting directed research in this area is imperative as the potential public health implications of climate change-induced weakening of the immune system at both individual and population levels are profound. This is particularly relevant for the already vulnerable children of the developing world, who will bear a disproportionate burden of future adverse environmental and geopolitical consequences of climate change.

## 1. Introduction

There is now unequivocal evidence that Earth’s climate is warming, greenhouse gas concentrations have increased, snow and ice cover have decreased and the sea level has risen. It is very likely that these changes are human induced [1]. Climate change will affect the fundamental determinants of human health including “water, air, food quality and quantity, ecosystems, agriculture, livelihoods and infrastructure” [2] (p. 393). The full range of potential consequences of climate change for human health is unknown, but there is agreement that the overall impact will be negative [3]. Some population subgroups are particularly vulnerable—children, the elderly, and the poor—and are likely to be disproportionately affected [4].

In the Global Burden of Disease assessment of 2000, 150,000 deaths worldwide were attributed to climate change [5] with 88% of the disease burden caused by loss of life or well-being in childhood. Most research into the human health effects of climate change has focussed on specific exposures (e.g., vector-borne diseases, extreme weather events), or occasionally on specific outcomes (e.g., asthma [6], pre-term birth [7]). Yet indirect effects, such as on food security and quality and on mental health, may also be important, but more difficult to predict or quantify. An alternative to consideration of specific direct and indirect exposures, or disease outcomes, is to examine potential risks from climate change to the fundamental systems underlying the human interaction with the environment. The immune system is an interface across which many climate change sensitive exposures will affect health outcomes. Exposures *in utero* and early childhood may tune—or “program”—the immune system toward an allergic or autoimmune propensity [8,9] with implications for later life, while robust immune responsiveness is required for protection from childhood infectious diseases and the success of vaccination programs. Particularly in the developing world context where conditions often favour disease (*i.e.*, undernutrition, overcrowding, poor sanitation), the immune system’s role in maintaining the primacy of the “healthy state” is continually challenged.

Here we examine mechanisms by which climate change-related phenomena could affect human immune function, with a focus on implications for child health. Just as the adverse physical effects of climate change are predicted to weigh most heavily on developing nations, children are likely to also be the most vulnerable to systemic adverse effects on immune function. By way of background to the broader discussion, we begin by providing a brief primer of the developing immune system to highlight particular periods and areas of vulnerability.

## 2. Developmental Immunology

The human immune system does not fully mature until after adolescence, thereby conferring increased risk of infection and illness and altered response to vaccination during the pre-adolescent period. The evidence to support this contention is compelling, with infection responsible for over two-thirds of deaths amongst children younger than 5 years [10,11]

Cells of the nascent human immune system emerge within weeks of conception faced with the multiple concomitant challenges of providing defence against infection at the placental interface, avoiding development of (allo-) immune reactions against maternal antigens and managing the transition from a sterile placental environment to the antigen-rich external world [12,13]. The gestational period is therefore a time of flux and vulnerability for the immature immune system.

The neonatal period is characterised by a reliance on the innate immune system (e.g., Toll like receptors, neutrophils, dendritic cells) given the limited exposure of the nascent adaptive immune system (comprised of antibodies (immunoglobulins, Ig), and memory T and B cells that drive cell-mediated immunity) to antigens *in utero.* Neonatal innate immune responses are not robust, giving rise to potentially serious infections with pathogens such as Group B Streptococcus, *Listeria monocytogenes* and Respiratory Syncytial Virus (RSV) [12]. High levels of maternal antibody (IgG) circulate within the neonate at birth offering passive protection from infection but these wane over the first 6 to 9 months of life; prematurity is associated with lower initial maternal IgG levels and thus greater vulnerability to infection. Babies who are breast-fed receive maternal IgA through breast milk and this offers passive protection of mucosal surfaces (e.g., gut, lungs). In terms of the infant’s own antibody production, endogenous IgM synthesis begins at birth, IgG is produced in useful amounts from 6 months of age and serum IgA levels rise slowly (20% of adult levels by 12 months). This combination of waning maternal antibodies and gradually development of infant antibodies results in a relative antibody nadir from 3 to 12 months with risk of infection by extra-cellular bacteria in particular [14,15]. In addition, antibody responses to thymus-independent antigens (e.g., polysaccharides) do not develop until late infancy (~24 months) leading to susceptibility to infection by encapsulated bacteria (e.g., *Streptococcus pneumonia*, *Neisseria meningitides*, *Haemophilus influenza*), that is responsible for most of the infection-related mortality amongst neonates and infants [16]. Appropriate cell-mediated immune function, essential for immune responses to intra-cellular infections (e.g., viral infection) develops by 12 months of age, with evidence of both skewed helper T cell (T_h_ cell) responses (CD4+) [17] and immature cytotoxic (CD8+) T cells prior to this age [15]. The development of the immune system continues through adolescence under the influence of surging levels of sex hormones. This period is associated with an increased incidence of auto-immune diseases [15]. 

## 3. Clinically Significant Immune Dysfunction: Breaching the Threshold

Disease can occur from both overactivity (up-regulation) of the immune system and immune suppression (down-regulation). Allergy is thought to result from up-regulation of T_h_2 cell mediated pathways, whereas some autoimmune diseases appear to be the result of up-regulated T_h_1 responses, resulting in diseases such as Type 1 diabetes, scleroderma and multiple sclerosis. The immune system defends against microbial pathogens and aberrant host cells, so that down-regulation may increase vulnerability to infection and disease (*i.e.*, cancer).

Impairment of specific immune components predisposes to specific types of infection and disease. For example, individuals with very low CD4+ (T_h_) cell counts (*i.e.*, HIV/AIDS) are at much higher risk of opportunistic infections such as *Pneumocystis jiroveci* pneumonia, mycobacterial infection and toxoplasmosis than those with a higher number of CD4+ T cells [18]. Primary antibody deficiency states, manifest by low or absent levels of circulating immunoglobulins (*i.e.*, IgG subclass deficiency, IgA deficiency or common variable immunodeficiency), are associated with recurrent respiratory and gastrointestinal infections of varying severity [14]. Additionally, studies of cancer patients [19] and acutely ill surgical patients [20] have shown that impaired cell mediated immunity is associated with poorer prognosis and higher mortality. 

**Figure 1:**
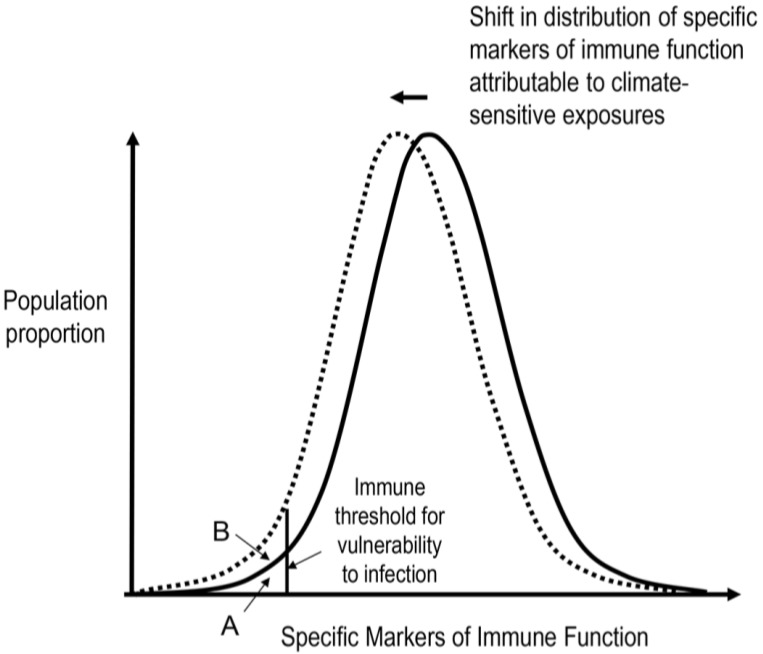
The influence of climate change-sensitive exposures on specific markers of immune function at a population level. Climate change related phenomena can subtly alter specific parameters of immune function at an individual level. Exposures having a broad impact at a population level could result in an increased proportion falling below critical immune thresholds that guard against infection and disease.

At an individual level, it is these profound impairments of immune function that are associated with significant (*i.e.*, clinically manifest) health consequences. However, at a population level, less profound impairment of specific immune processes occurring with high prevalence may manifest as an increased incidence of infection (e.g., influenza, otitis media or the common cold) [21] and reduced vaccine effectiveness [22] (Figure 1). For vulnerable sub-populations—those at extremes of age, ill and in the developing world—these outcomes may be even more pronounced. We postulate that a number of exposures that are affected by climate change may subtly impair aspects of a child’s immune response or critical events in the immune system’s development, thereby altering the distribution of vulnerability within the population.

## 4. How Can Global Climate Change Affect the Function of a Child’s Immune System?

**Figure 2:**
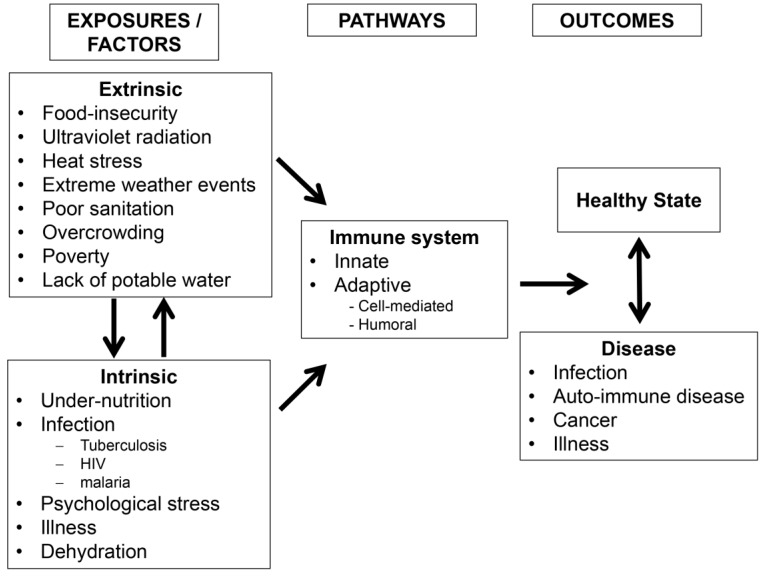
The developing immune system can be influenced by extrinsic and intrinsic exposures and physiological states that can lead to adverse health outcomes.

Given the complexity of the development, maturation and overall structure of the human immune system, there are various ways in which a change in climatic conditions can influence immune function. Some of those influences are mediated by climatic impacts on environmental systems and processes; others by influences on culture and human behaviour (Figure 2). Notably, for already vulnerable children of the developing world, many of these factors are compounded, and their effects may be additive or multiplicative.

The role of under-nutrition in late foetal life and in early childhood has been well documented. Under-nutrition that is wholly or partly due to climate change can arise not only from adverse effects on food yields and storage, especially cereal grains, but from the chronic diarrhoeal disease that characterizes childhood experience in many of the world’s poor, crowded and unhygienic “informal housing” settlements where food and water is often faecally contaminated and domestic hygiene is deficient. Human-driven climate change is projected to impair crop yields in many regions of the world, especially South Asia and much of Sub-Saharan Africa (SSA), as a consequence of changes in temperature, rainfall and soil moisture [23] and is expected to also occur because of damage from increases in extremes of weather and changes in patterns of infestations and infections—in plants and livestock.

Many infectious diseases are sensitive to small shifts in climatic conditions, including vector-borne infections spread by mosquitoes and ticks. In poor and vulnerable populations, cholera outbreaks are more likely to occur during both extreme flooding and droughts [24]; so too are various other types of gastroenteritis. Climate change will affect the geographic range and seasonality of mosquito-borne diseases such as malaria, dengue fever, West Nile virus and Japanese encephalitis, reducing the incidence in some populations, increasing it in others and introducing it to other immunologically naïve populations [25]. Changes in the timing and rate of exposure of infants to infectious agents, whether or not they produce clinical disease, influences the maturation of the young immune system including toward atopic and auto-reactive phenotypes [26,27].

Children that are displaced by enforced relocation or by refugee flows in response to increasing climatic adversity—food shortages, physical hazards, loss of arable or habitable coastal land, *etc.*—are very often exposed to both chronic food deprivation and to a range of infectious agents encountered during crowd movement and in congested and unhygienic refugee camps [28].

Climate change and its impacts will influence physiological and psychological stress on many young human bodies and minds. Reflecting the immune system’s complexity, there are many neuro-hormonal axes and pathways via which acute or chronic stress levels can affect aspects of immune functioning [29]. Psychological depression is a well-recognised modulator of immune function [30]. Clearly evident in many studies of animal models, though less well demonstrated in humans, chronic heat stress also impairs aspects of the immune response [31,32].

Chronic background stress will impinge on many families and their children, in all regions of the world, as climate change conditions become more disruptive and severe. Strazdins and Skeats [33] remind us that: “children’s wellbeing will be affected by expected economic, social and cultural impacts of climate change. If, as forecast, climate change results in food and water scarcity then there will likely be an increase in the number of families living in poverty. In rural and remote areas, some families will lose their livelihood. This in turn may lead to forced internal migration: family separations where one parent moves to earn income or family dislocation where the whole family moves, especially likely for rural families or families caught up in a climate change related natural disaster.” Those authors also point out, however, that there has been little research specifically on the childhood stress experiences of climate change and their biological, health and behavioural consequences.

In the following sections, we discuss three specific exposures, namely under-nutrition, psychological stress and ultraviolet radiation exposure, for which there is reasonable likelihood of effects on children’s immune systems, and that are likely to be altered under climate change conditions. 

## 5. Under-Nutrition, Climate Change and Immune Function

### 5.1. Under-Nutrition and Human Immune Function

Under-nutrition is the most common cause of secondary immune suppression in children globally and may result from inadequate intake of macro- (carbohydrates, protein, fat) or micro-nutrients (essential vitamins, minerals) [34,35]. Macro-nutrients provide the energy required to carry out daily activities and to build and repair tissues. Micronutrients are required for the optimal functioning of cellular processes and metabolic pathways. 

Macro-nutrient deficiency or “protein-energy malnutrition” (PEM) results in a generalized depression of immune function, particularly in young children [36,37]. Within the innate immune system, complement activation, phagocytosis and cytokine production are all depressed [34,38]. Lymphatic organs (spleen, thymus and lymph nodes) undergo atrophy [39]. Function of the adaptive immune system can be impaired, with reductions in antibody secretion and affinity to antigen. This has implications for the efficacy of vaccinations (e.g., tetanus, measles) [40,41]. Severe PEM can lead to leucopenia (decreased number of white blood cells), decreased T_h_ cell (CD4+) and cytotoxic T cell (CD8+) numbers, as well as a reduced CD4+:CD8+ ratio—considered an important correlate of susceptibility to infection [34,35].

Micro-nutrient under-nutrition often accompanies PEM, but can occur as an isolated deficiency (*i.e.*, of iron, vitamin A or zinc). The extent and nature of immune dysfunction depends on the specific micro-nutrient involved. A deficit of zinc, for example, is associated with lymphoid atrophy and decreased delayed-type hypersensitivity skin test responses and has been also associated with increased mortality and morbidity from infection in animal models *Enterovirus*, coxsackie B, *Listeria monocytogenes*) [34]. Of the micro-nutrients, deficiencies of vitamins (A, C, E, B6), selenium, zinc, copper, iron and folic acid are associated with impaired immune function and/or increased rates of infection in humans [34,36].

### 5.2. Climate Change and Under-Nutrition

The overall nutrition of a population, and especially of its vulnerable sub-populations, is dependent on the state of “food security”, which is a composite of food *availability*, physical and economic *access* to that food and *appropriate utilization* of nutrients [42]. Climate change can negatively affect any of these factors.

There are modelled predictions that the temperature and rainfall (hence, soil moisture) changes that are central to climate change may increase food production in some regions of the world [43]. There may also be a positive “fertilizer” effect on agriculture due to increased atmospheric CO_2_ [44]. However, and particularly in areas of current vulnerability to food insecurity such as SSA and Asia, the modelled impacts of climate change on food yields suggest greatly reduced yields. Decreases in crop yields are projected to occur as a result of direct thermal stress on crops, altered timing of seasons, reduced available arable land and water for agriculture, increased soil salinity and diminished biodiversity [42,43]. An altered frequency of extreme weather events will also affect future yields.

The most recent IPCC assessment report rates as “very likely” that climate change will have an overall negative effect on major cereal crop yields across Africa, with strong regional variability [23]. Nelson *et al.* [45] undertook simulations of all sub-Saharan regions and demonstrated consistently negative effects of climate change on major cereal crops, ranging from 2% for sorghum to 35% for wheat by 2050 (under an “A2” scenario). Another study projected that wheat growing could disappear from the African continent by 2080 [46]. For south Asia, home to the greatest number of food insecure children, a large systematic review and meta-analysis of original data publications demonstrated a crop yield reduction of −16% for maize and −11% for sorghum by the 2050s [47]. Notably, this study did not project a mean change in rice production for south Asia over this time.

Access to food under climate change scenarios may be adversely affected as a result of market inequities, pricing barriers or employment insecurity (especially for landless agricultural labourers). It has been estimated that global cereal prices will increase by more than three-fold by the 2080s (compared with current day market prices) [43]. If incomes do not rise commensurately, this will have obvious negative down-stream effects on the children of poor families. Furthermore population displacement due to climate change, (e.g., sea level rise, extreme weather events or conflict) may limit access to nutritious foods both through affordability and lack of availability of familiar foods.

Appropriate utilization of food can be adversely affected by climate change via conditions leading to decreased absorption of nutrients (e.g., diarrheal illness, parasitic gut infection), increased energy requirements (e.g., concomitant infections, increased physical work load) and/or unsafe food preparation (e.g., disrupted water and sanitary systems) [42]. Several studies have shown an association between rising ambient temperature and increased rates of infectious diarrhea (with specific pathogens such as *Salmonella* [48] and *Campylobacter* [49]) and non-specific diarrheal illness [50]. Extreme weather events can also overwhelm sanitation and water management systems, particularly in developing areas where infrastructure is often inadequate. Large outbreaks of cholera in Asia and SSA, for example, have occurred due to contamination of water supply following flooding episodes [51].

### 5.3. Under-Nutrition as a Mediator of Climate-Change Induced Immune Suppression

An estimated 26% of the world’s children are stunted due to severe chronic under-nutrition [52]. Approximately one third of the burden of disease in children is currently attributable to under-nutrition [53]. Although the overall population at risk is projected to significantly reduce during the 21st century due to socioeconomic development, it is likely that this progress will be uneven amongst regions of the developing world and slowest in the next few decades. Lloyd *et al.* [54] developed a model for estimating future under-nutrition amongst children, taking into account food production and access, and socioeconomic factors (e.g., female literacy, healthcare access, economic growth) for regions in south Asia and SSA. They concluded that climate change would lead to an increase of 1%–29% in moderate stunting in 2050 compared with a reference scenario (of no climate change). Severe stunting was projected to increase by 23% in central SSA and 62% in South Asia compared with the reference scenario.

We postulate that under-nutrition associated with climate change will impair immune function in a non-uniform manner with already vulnerable populations expected to fare disproportionately poorly. It has been recently estimated that approximately 20% of children under 5 years in low-to-middle income countries are underweight (weight-for-age less than two standard deviations (SD) from the population mean) [53]. Additionally in this study, data from eight low-income countries (Ghana, Guinea Bissau, Senegal, Philippines, Nepal, Pakistan, India and Bangladesh) were analysed for disease risks associated with childhood under-nutrition. After adjusting for non-nutritional determinants of infection and mortality, for severely underweight children (<3 SD from mean), the odds ratios for overall mortality (9.7) and disease-specific mortality (diarrhoeal illness (9.5), pneumonia (6.4), measles (6.4) and malaria (1.6)) were significantly raised. Thus it is likely that the future effects of climate change on immune function, mediated by reduced food security, will contribute to ongoing vulnerability to infection, particularly for children in the developing world.

## 6. Psychological Stress, Climate Change and the Immune System

### 6.1. Psychological Stress and Immune Function

Psychological stress occurs when events or demands overwhelm an individual’s perceived capacity to cope, eliciting a physiological stress response [55]. While acute time-limited stressors, such as mental arithmetic or public speaking can enhance immune parameters, particularly those associated with innate immunity, longer term stressor exposure tends to depress cell-mediated immunity (T_h_1), upregulate T_h_2-associated cytokines and antibodies to latent viruses (e.g., EBV), and depress innate immunity (reviewed in [29]).

There is increasing, but still limited, research on the effect of stress on immune function in children. Parent-reported perceived stress and depressive symptoms [56] and a harsh family climate [57] were associated with a pro-inflammatory profile in children and adolescents (respectively), while higher perceived self-efficacy in children aged 7–10 years was associated with an anti-inflammatory profile, *i.e.*, lower IL-6 concentrations [58]. In the latter study, depression was associated with increased risk of febrile illness only in older girls. This is consistent with stages of particular sensitivity of the immune system to psychological factors, during the prenatal period, in infancy and in the pubertal transition, although most of the current evidence is for neuro-behavioural outcomes [59] and HPA reactivity [60,61]. Another recent study involving healthy 5 year old children from low and high psychological stress environments, showed evidence of high stress leading to an imbalance in the immune response and a predilection to reactivity to self-antigens [62], possibly leading to autoimmune disorders. Impacts appear to be cumulative across different domains such that those most at risk from environmental stressors are those already under socioeconomic or psychological stress [63,64] with long term effects on health [65].

There is emerging evidence that stress-related effects on immune function can have clinical consequences. In a recent study of children aged 0–17 years, there was a higher incidence of type 1 diabetes, an autoimmune disorder, in regions of Israel that were attacked in the Second Lebanon War compared to other regions and to pre-war incidence, after taking account of family history of disease, age, sex, and season of diagnosis [66].

### 6.2. Psychological Stress and Climate Change 

Climate change may have an impact on mental health in several ways, although most research has focused on outcomes in adults [67]:

#### I. Acute traumatic stress following an extreme event:

In the aftermath of the devastation wrought by Hurricane Katrina on the US Gulf Coast in 2004, one study showed a marked increase in the rates of psychological illness amongst affected populations [68]. Sixty-nine percent of the study population described symptoms of depression or low mood, with 50% meeting clinical criteria for major depression. Rates of attempted, and completed, suicide were 78 and 14 times the baseline rate respectively. Similarly, one year after the 1999 “super-cyclone” which struck the Indian state of Orissa, a significant proportion of children and adolescents were diagnosed with post-traumatic stress disorder (31%) and syndromal depression (24%) [69].

#### II. Disruptions to social, economic and environmental determinants that promote mental well-being

Mental well-being is associated with stability in food supply, housing, family and community, an adequate health infrastructure and a strong economy [70,71]. Global climate change has the potential to disrupt some or all of these conditions, and thus the potential to undermine the long-term psychosocial well-being of affected populations [72]. For example, it has been estimated that over 200 million persons may be forced to leave their place or country of residence by 2050 due to a combination of climate change-related shoreline erosion, coastal flooding, desertification, agricultural change, natural disasters, government policy or geopolitical conflict [73]. Studies have shown that disaster related relocation is a strong predictor of psychological difficulties [74]. In rural Australia, prolonged drought conditions have affected many historically fertile areas. A 2004 study of adolescents from drought-stricken areas showed that they were aware of the impacts of drought on their families and communities, but did not report higher levels of emotional distress. However a follow-up four years later, in the face of ongoing drought, showed significantly higher levels of emotional distress, with thematic analysis noting themes of grief, loss and concern about the impacts of climate change [75]. Additionally, an association between increased suicide rate and step-downs in inter-annual rainfall has been demonstrated [76].

#### III. Anxiety and fear for the future in a Climate Changed World

This last category refers to the psychological reaction of individuals to the stream of often dire predictions regarding the consequences of global climate change emanating from peers, teachers and the scientific and popular media. Over time, this barrage of unsettling, overwhelming and threatening information may lead to a state of chronic low-grade anxiety, fear or hopelessness. Children may be a particularly vulnerable group in this regard. For example, schoolchildren’s perceptions at the height of the Cold War were characterized by despair and loss of motivation [77]. An Australian study of 600 children (aged 10–14 years) reported that 44% were concerned about the future impact of climate change, 31% were worried they would have to eventually fight in a war and 25% believed the world would end before they got older [78]. How children cope with these stressors is dependent on their individual levels of vulnerability and resilience, which are closely linked to physical health, household dynamics, parental coping ability and availability of social support [79]. Climate change related phenomena can again affect these dynamics.

### 6.3. Psychological Stress as a Mediator of Climate Change Induced Immune Dysfunction

Following extreme weather events, where numerous stressors may already be affecting immune function (e.g., overcrowding, exposure to temperature extremes, sleep disturbance), prolonged psychological stress can further modulate the immune response. For example, 33% of Florida residents affected by Hurricane Andrew suffered from post-traumatic stress disorder (PTSD) in the first four months (76% had at least on symptom of PTSD), and this was associated with lower natural killer cell activity, a marker of innate immune function [80]. Indeed, in the overall affected study population, there was a significant decrease in natural killer (NK) cell functional activity and T cell lymphocyte (CD4+ and CD8+) numbers compared with controls.

For vulnerable populations, such as children and communities in the developing world with limited adaptive capacity, the impact of climate change associated with psychological stress is of particular concern.

## 7. Ultraviolet Radiation, Climate Change and Immune Function 

### 7.1. Ultraviolet Radiation

The vast majority of human exposure to ultraviolet radiation (UVR) is from sunlight. UVR is required to initiate vitamin D (an essential steroid hormone) synthesis from precursors in the skin [81]. UVR is arbitrarily divided into three wavelength bands: UVA, UVB and UVC. All incoming solar UVC and over 90% of UVB is absorbed by stratospheric ozone and other gases, such that the majority of UVR at Earth’s surface is UVA [82]. However, UVB is more biologically effective than UVA and remains the most important contributor to UVR effects on human health [83]. Factors which may increase ambient UVR levels (and the relative proportions of UVB and UVA) include: high altitude, low latitude, time around midday, clear or partly cloudy skies and surrounding reflective surfaces (*i.e.*, snow, water) [84]. At an individual level, the received personal dose of UVR depends on the duration and timing of periods spent outside, type of clothing worn, use of sunglasses and sunscreen, and skin pigmentation. Children typically receive ~3% (2%–4%) of the total ambient UVR, which is similar to indoor-working adults, while outdoor working adults receive, on average, around 10% of ambient UVR [85]. 

### 7.2. UV Radiation and the Human Immune System

Numerous studies have revealed the local and systemic immunosuppressive effects of UVR on the human immune system [86,87]. Underlying UVR induced immune-modulating mechanisms that have been demonstrated include:

- Suppression of the activity of cutaneous antigen presenting cells (APC) (leading to migration away from skin, and impaired interaction with T cells in the lymph node);
- Promotion of specialised regulatory T cells (T_reg_) which produce immune inhibitory cytokines (particularly IL-10);
- Inhibition of cytotoxic and memory T cell production and function.
- Cutaneous production of vitamin D, of which the active form (1,25 hydroxyvitamin D_3_) has been shown to down-regulate cell-mediated immune function processes (e.g., enhanced T_reg_ cell function) and promote innate immune processes (e.g., anti-microbial peptide production) [88].

Overall, the effect of exposure to UVR is one of down-regulation of cell mediated (T_h_1) processes and promotion of a regulatory environment within draining lymph nodes. 

Animal model and human clinical studies have also shown an association between UV irradiation and increased incidence of skin tumours and infection. In animal models, UV irradiation has resulted in reduced immune responses following infection with a range of pathogens, including: *Listeria monocytogenes*, *Mycobacterium leprae*, *Mycobacterium bovis* (BCG), *Trichinella spiralis*, *Leishmania*, *Borrelia burgdorferi*, *Plasmodium chabaudi* and *Candida albicans* [89,90]. In humans, reactivation of herpes simplex virus (HSV) and human papilloma virus (HPV) infections appear to be related to UVR exposure, through viral-tropism and immunosuppression [91].

There is evidence, albeit limited, suggesting that increased UVR exposure can cause decreased vaccine effectiveness [92]. Given the enormous public health impact if shown to be clinically relevant, this area is the subject of ongoing research. Significant suppression of cell mediated and humoralimmunity occurred following experimental UVB exposure in mice recently vaccinated with hepatitis B [93]. Cell mediated immune suppression (as measured by contact hypersensitivity reactions) was suppressed in similar experiments on healthy human subjects, though notably, there was no clinically significant reduction in the measured antibody response [94]. There was however, significant variation in immune response following UVR exposure dependent on specific cytokine gene polymorphisms [95].

A number of observational studies have examined the association between UVR exposure and efficacy of vaccination in paediatric populations [96,97,98,99]. These studies have varied by geographic region, vaccine type and study design including time-points for measuring immune end-points and surrogate markers of UVR exposure (e.g., season of vaccination, latitude of residence). Despite these differences, the overall picture is one of lower vaccine effectiveness in regions or seasons with high ambient UVR exposure.

Studies have shown decreasing prevalence of autoimmune diseases (such as Type 1 diabetes, multiple sclerosis and connective tissue disorders) associated with higher levels of surrogate markers of solar UVR exposure [100,101]. This has been represented as further evidence of the influence of UVR exposure on suppressing cell-mediated responses, directly or indirectly via vitamin D-mediated mechanisms [102].

### 7.3. Climate Change and UV Radiation

Global climate change is expected to influence future personal UVR exposure via altered atmospheric conditions and changing behavioural, clothing and outdoor activity patterns. Changing atmospheric dynamics, including interactions between ozone, ozone-depleting gases (e.g., CFCs) and greenhouse gases are complex and have significant regional variation [103]. A study of future biologically relevant (“erythemal”) UVR exposure projected reductions of 9% in northern high latitudes, and increases by 4% in the tropics and 20% in southern high latitudes in late spring and early summer [104].

Ground level UVR is also strongly influenced by cloud cover, which will likely be altered in unpredictable ways under climate change conditions. Though heavy cloud cover will likely diminish the amount of UVR reaching Earth’s surface, partly cloudy skies can lead to large enhancements due to the effect of scattering [105]. Taking into account cloud cover changes, recent modelling predicts a decrease in ambient erythemal UV radiation of 10% at northern high latitudes and an increase of 3%–6% at low latitudes [106].

A more significant impact on personal UVR dose may arise through changes in behaviour and clothing patterns. That is, in warmer conditions, people tend to spend more time outdoors and wear clothing that exposes more skin to sunlight. This may particularly be the case for populations at mid to high latitudes. A British study has shown that primary and secondary schoolchildren living at lower latitudes spend more time outside on weekends than those from higher latitudes, which may be explained in part by geographic differences in ambient temperature and climate [107].

### 7.4. Ultraviolet Radiation as a Mediator of Climate Change-Induced Immune Dysfunction

The likely overall effects of climate change on predicted levels of UVR at Earth’s surface are for an increase in current latitudinal gradients—*i.e.*, higher levels at low latitudes where they are already high, and lower levels at high northern latitudes where they are already low [106]. If these predictions are verified (noting the uncertainties in the assumptions used in the models), they could have significant consequences for UVR-related health outcomes at a population level [108].

In many parts of the world, particularly in temperate zones, we postulate that personal UVR exposure will increase due to increased outdoor activity and less clothing coverage due to warmer weather. This may have beneficial effects, for example in decreasing incidence rates of T_h_1-mediated autoimmune diseases such as type 1 diabetes and multiple sclerosis, if a causal association truly exists with UVR dose. An interesting recent Australian study showed an inverse association between maternal ambient UVR exposure during the first trimester of pregnancy and the risk of multiple sclerosis in the offspring [109]. A similar pattern of results was seen in a Northern hemisphere study [110], suggesting the influence of UVR exposure (perhaps mediated by vitamin D) at critical times of *in utero* immune system development. Improved vitamin D status (consequent upon higher UVR dose) may also benefit bone and muscle health, possibly reducing risk of certain cancers [111], cardiovascular, rheumatic and other disorders [112].

However, there is considerable potential for adverse effects caused by excessive UVR dose, particularly in developing countries. Here individuals may not be able to change the duration of their time outside under pressures to maintain food production or other activities. Higher levels of ambient UVR and temperature may individually or in combination impair the immune response to vaccination and increase risk of infections. For example, a risk assessment study estimated that exposure to just 90 min of midday, mid-latitude sunshine in the summer months in sensitive, non-adapted individuals, would lead to a 50% reduction in specific cell-mediated immune responses to *Listeria monocytogenes*, an intra-cellular bacterium [113]. Outbreaks of measles infection amongst previously vaccinated children in north India has also been tentatively linked to excessive UVR exposure [99].

## 8. Conclusions and Implications

The immune system comprises a set of complex, interacting, mechanisms through which the human body protects itself against microbial assault, cancer and illness. The gestational period and early childhood are especially vulnerable phases, both because the immature immune system is sensitive to a range of external exposures and stresses, and because the consequences of any childhood deficits in full immune competence can become a life-long liability in relation to health and disease.

Here we have focused on undernutrition, psychological stress, and ultraviolet radiation as possible mediators of the effect of climate change on immune-related health risks in childhood. Given the complexity of the climate system and the manifestations of changes in it, there are likely to be many others. Though the magnitude of deterioration in immune function for any given climate change-sensitive variable may be small, their combined effects on whole populations may lead to significant protective clinical thresholds being breached. This will be particularly relevant for the already vulnerable children of the developing world, who will likely bear a disproportionate burden of future adverse environmental and geopolitical consequences of climate change.

Demonstration of a causal link between climate change and health outcomes is enormously challenging. Health impacts plausibly linked to climate change have been well described [25] but to definitively establish that these effects have been, at least partially, mediated via climate-induced changes in immune function is fiendishly difficult given the confounding influence of climate on pathogens and the environment. Animal models exist for the impact of environment on immune function, and many observations link climate and environment to human disease, but there are no studies of important childhood diseases that include measurement of both climate exposures and measurement of immune mediation of these exposures in influencing disease incidence.

Conducting further well directed research in this area therefore is imperative: the potential public health implications of a weakened immune system at both individual and population levels are profound.
